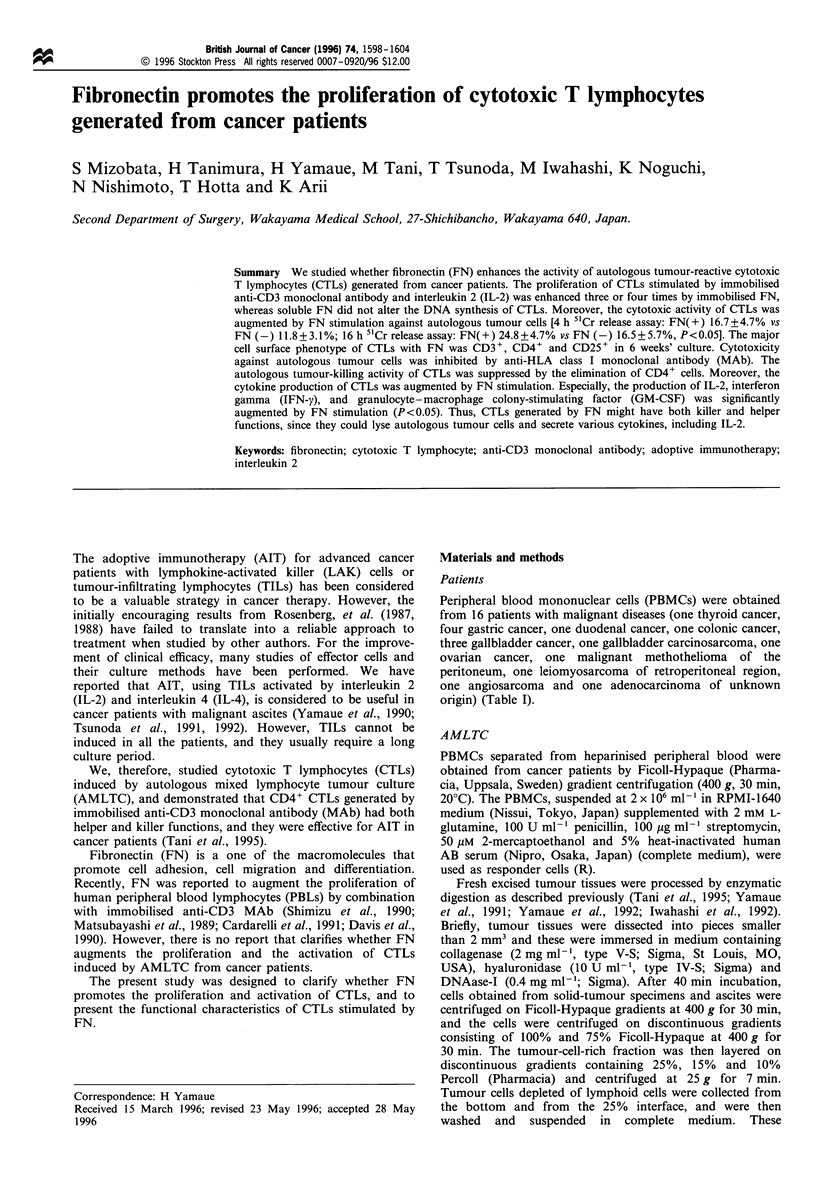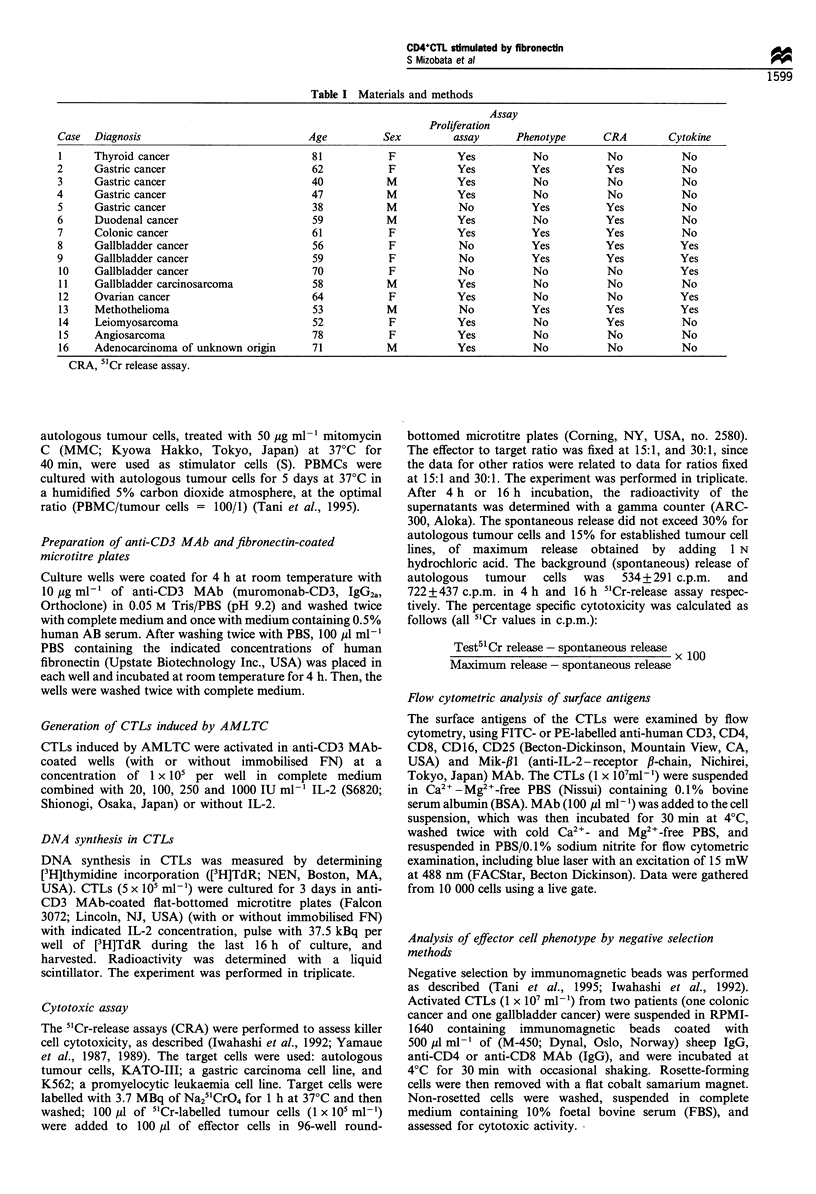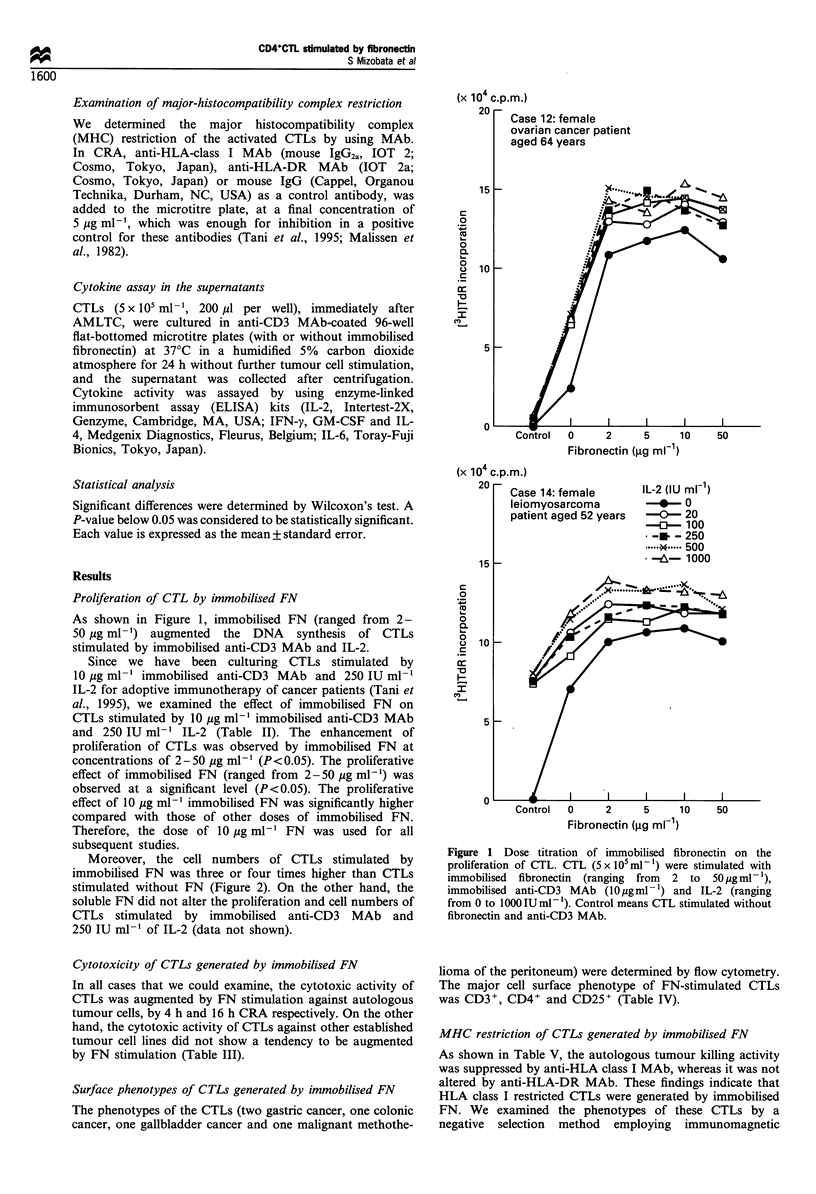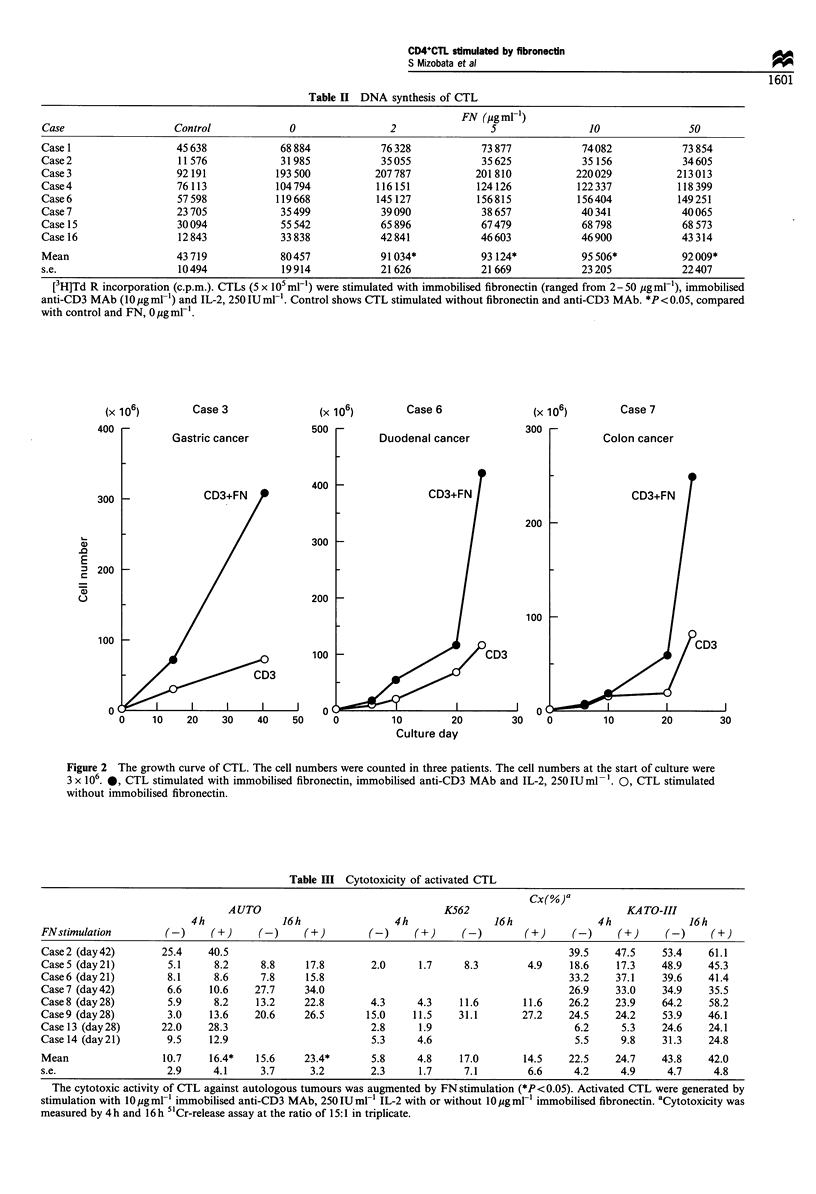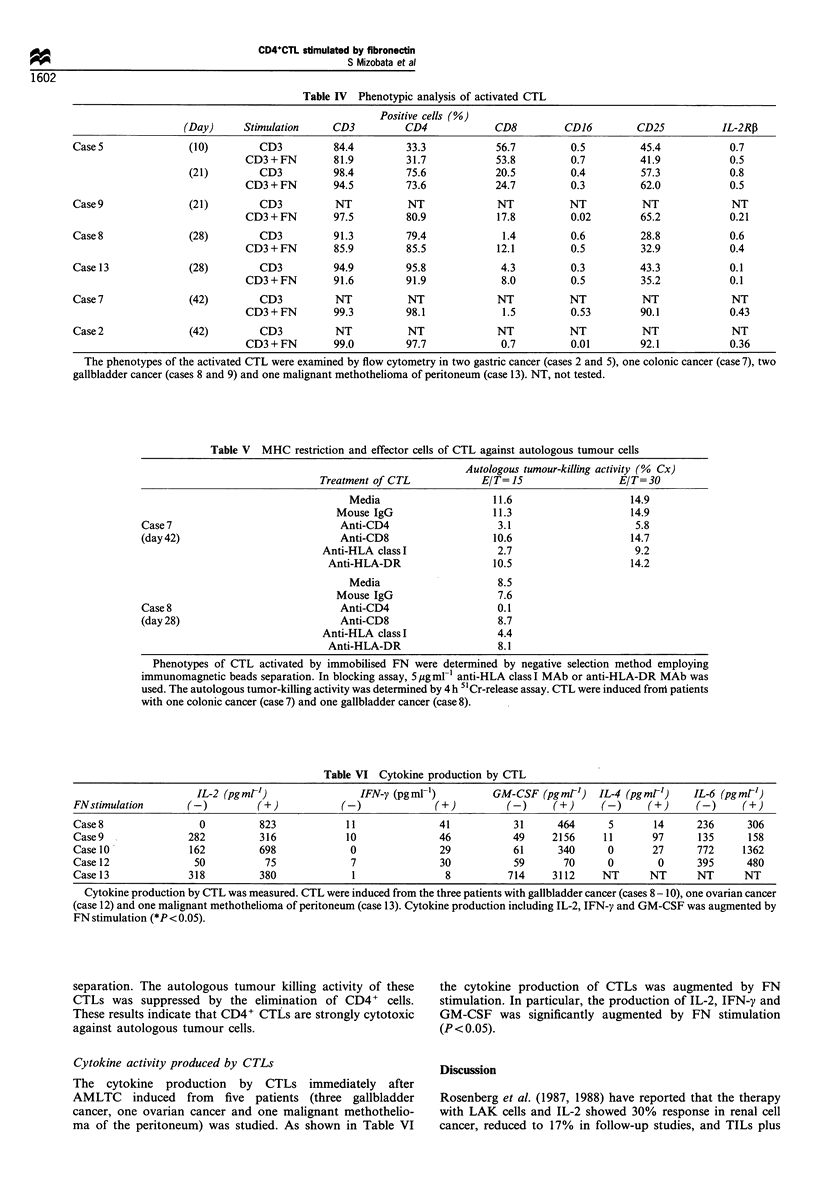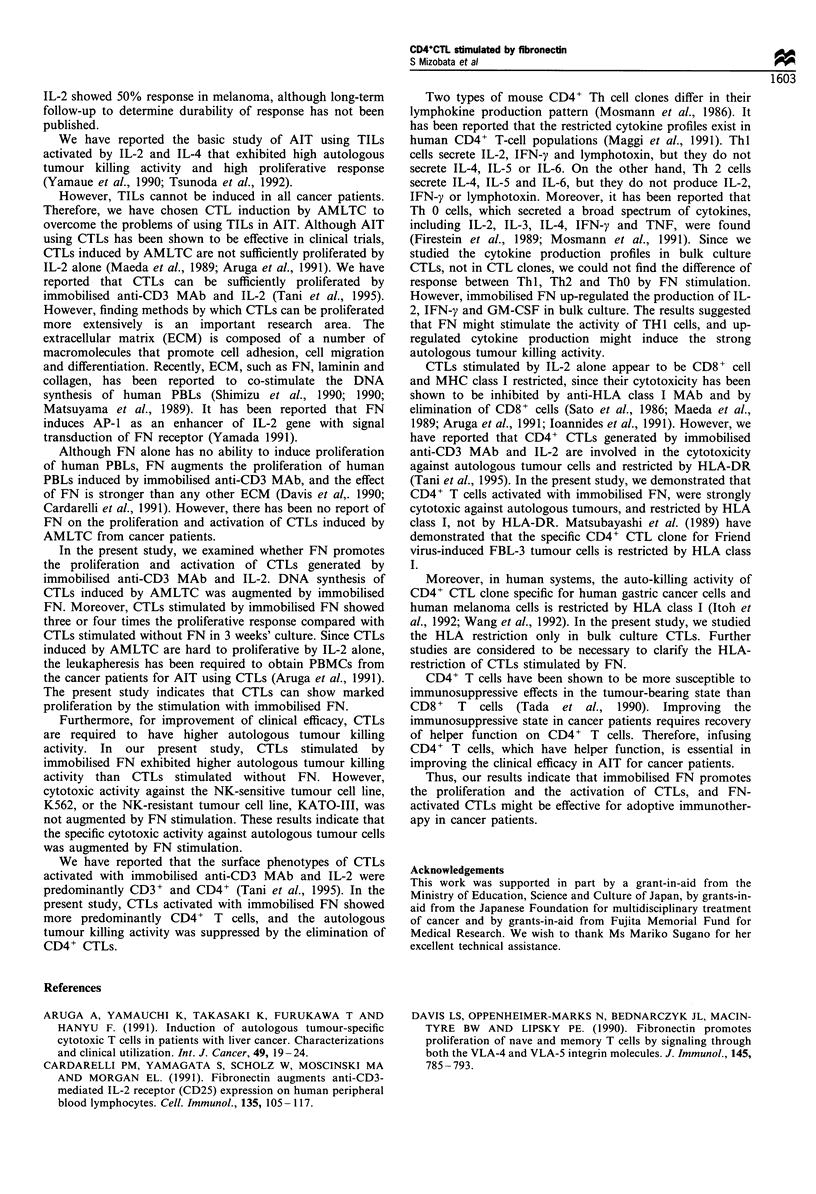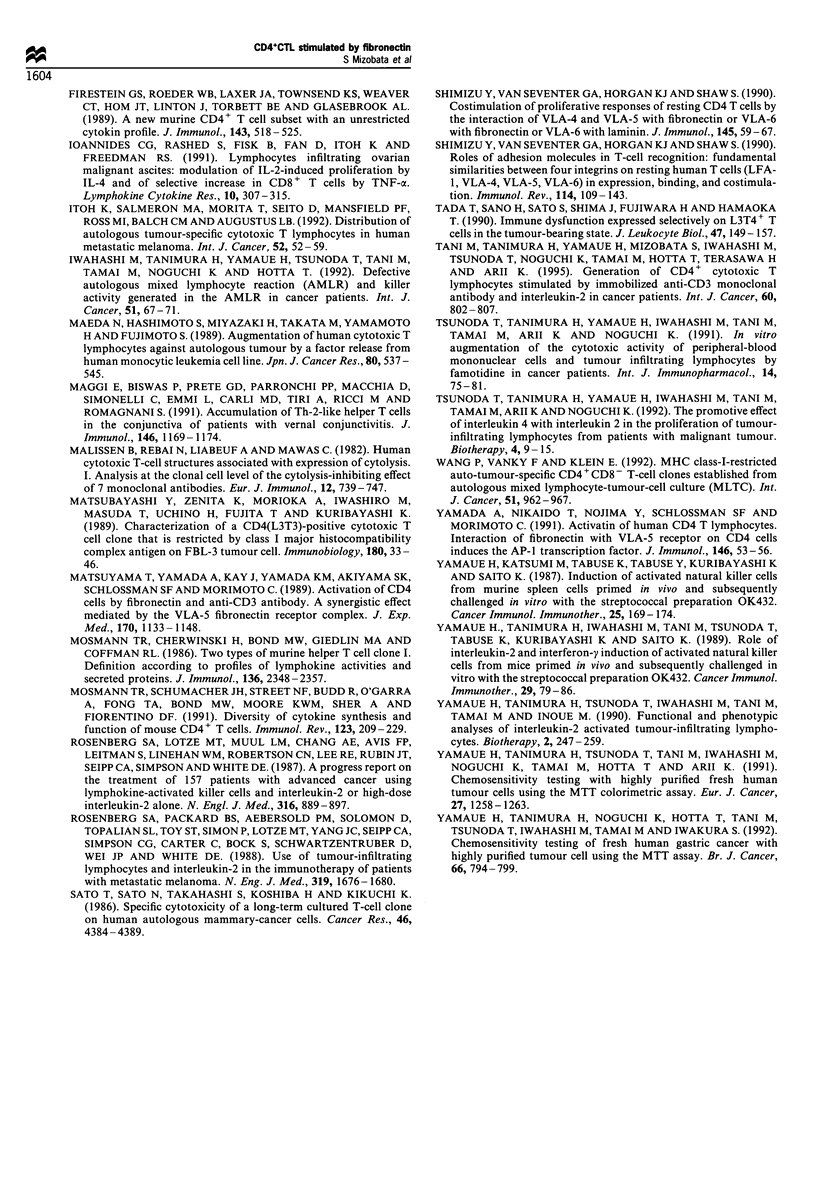# Fibronectin promotes the proliferation of cytotoxic T lymphocytes generated from cancer patients.

**DOI:** 10.1038/bjc.1996.595

**Published:** 1996-11

**Authors:** S. Mizobata, H. Tanimura, H. Yamaue, M. Tani, T. Tsunoda, M. Iwahashi, K. Noguchi, N. Nishimoto, T. Hotta, K. Arii

**Affiliations:** Second Department of Surgery, Wakayama Medical School, Japan.

## Abstract

We studied whether fibronectin (FN) enhances the activity of autologous tumour-reactive cytotoxic T lymphocytes (CTLs) generated from cancer patients. The proliferation of CTLs stimulated by immobilised anti-CD3 monoclonal antibody and interleukin 2 (IL-2) was enhanced three or four times by immobilised FN. whereas soluble FN did not alter the DNA synthesis of CTLs. Moreover, the cytotoxic activity of CTLs was augmented by FN stimulation against autologous tumour cells [4 h 51Cr release assay: FN(+) 16.7 +/- 4.7% vs FN (-) 11.8 +/- 3.1%; 16 h 51Cr release assay: FN(+) 24.8 +/- 4.7% vs FN (-) 16.5 +/- 5.7%, P<0.05]. The major cell surface phenotype of CTLs with FN was CD3+, CD4+ and CD25+ in 6 weeks' culture. Cytotoxicity against autologous tumour cells was inhibited by anti-HLA class I monoclonal antibody (MAb). The autologous tumour-killing activity of CTLs was suppressed by the elimination of CD4+ cells. Moreover, the cytokine production of CTLs was augmented by FN stimulation. Especially, the production of IL-2, interferon gamma (IFN-gamma), and granulocyte macrophage colony-stimulating factor (GM-CSF) was significantly augmented by FN stimulation (P<0.05). Thus, CTLs generated by FN might have both killer and helper functions, since they could lyse autologous tumour cells and secrete various cytokines, including IL-2.


British Journal of Cancer (1996) 74, 1598-1604
© 1996 Stockton Press All rights reserved 0007-0920/96 $12.00

### Fibronectin promotes the proliferation of cytotoxic T lymphocytes generated from cancer patients

S Mizobata, H Tanimura, H Yamaue, M Tani, T Tsunoda, M Iwahashi, K Noguchi, N Nishimoto, T Hotta and K Arii

*Second Department of Surgery, Wakayama Medical School, 27-Shichibancho, Wakayama 640, Japan.*

**Summary** We studied whether fibronectin (FN) enhances the activity of autologous tumour-reactive cytotoxic T lymphocytes (CTLs) generated from cancer patients. The proliferation of CTLs stimulated by immobilised anti-CD3 monoclonal antibody and interleukin 2 (IL-2) was enhanced three or four times by immobilised FN, whereas soluble FN did not alter the DNA synthesis of CTLs. Moreover, the cytotoxic activity of CTLs was augmented by FN stimulation against autologous tumour cells [4 h $^{51}Cr$ release assay: FN(+) 16.7±4.7% *vs* FN (−) 11.8±3.1%; 16 h $^{51}Cr$ release assay: FN(+) 24.8±4.7% *vs* FN (−) 16.5±5.7%, $P<0.05$]. The major cell surface phenotype of CTLs with FN was $CD3^+$, $CD4^+$ and $CD25^+$ in 6 weeks' culture. Cytotoxicity against autologous tumour cells was inhibited by anti-HLA class I monoclonal antibody (MAb). The autologous tumour-killing activity of CTLs was suppressed by the elimination of $CD4^+$ cells. Moreover, the cytokine production of CTLs was augmented by FN stimulation. Especially, the production of IL-2, interferon gamma (IFN-$\gamma$), and granulocyte-macrophage colony-stimulating factor (GM-CSF) was significantly augmented by FN stimulation ($P<0.05$). Thus, CTLs generated by FN might have both killer and helper functions, since they could lyse autologous tumour cells and secrete various cytokines, including IL-2.

**Keywords:** fibronectin; cytotoxic T lymphocyte; anti-CD3 monoclonal antibody; adoptive immunotherapy; interleukin 2

The adoptive immunotherapy (AIT) for advanced cancer patients with lymphokine-activated killer (LAK) cells or tumour-infiltrating lymphocytes (TILs) has been considered to be a valuable strategy in cancer therapy. However, the initially encouraging results from Rosenberg, *et al.* (1987, 1988) have failed to translate into a reliable approach to treatment when studied by other authors. For the improvement of clinical efficacy, many studies of effector cells and their culture methods have been performed. We have reported that AIT, using TILs activated by interleukin 2 (IL-2) and interleukin 4 (IL-4), is considered to be useful in cancer patients with malignant ascites (Yamaue *et al.*, 1990; Tsunoda *et al.*, 1991, 1992). However, TILs cannot be induced in all the patients, and they usually require a long culture period.

We, therefore, studied cytotoxic T lymphocytes (CTLs) induced by autologous mixed lymphocyte tumour culture (AMLTC), and demonstrated that $CD4^+$ CTLs generated by immobilised anti-CD3 monoclonal antibody (MAb) had both helper and killer functions, and they were effective for AIT in cancer patients (Tani *et al.*, 1995).

Fibronectin (FN) is a one of the macromolecules that promote cell adhesion, cell migration and differentiation. Recently, FN was reported to augment the proliferation of human peripheral blood lymphocytes (PBLs) by combination with immobilised anti-CD3 MAb (Shimizu *et al.*, 1990; Matsubayashi *et al.*, 1989; Cardarelli *et al.*, 1991; Davis *et al.*, 1990). However, there is no report that clarifies whether FN augments the proliferation and the activation of CTLs induced by AMLTC from cancer patients.

The present study was designed to clarify whether FN promotes the proliferation and activation of CTLs, and to present the functional characteristics of CTLs stimulated by FN.

Correspondence: H Yamaue
Received 15 March 1996; revised 23 May 1996; accepted 28 May 1996

### Materials and methods

### Patients

Peripheral blood mononuclear cells (PBMCs) were obtained from 16 patients with malignant diseases (one thyroid cancer, four gastric cancer, one duodenal cancer, one colonic cancer, three gallbladder cancer, one gallbladder carcinosarcoma, one ovarian cancer, one malignant methothelioma of the peritoneum, one leiomyosarcoma of retroperitoneal region, one angiosarcoma and one adenocarcinoma of unknown origin) (Table I).

### AMLTC

PBMCs separated from heparinised peripheral blood were obtained from cancer patients by Ficoll-Hypaque (Pharmacia, Uppsala, Sweden) gradient centrifugation (400 *g*, 30 min, 20°C). The PBMCs, suspended at $2\times10^6$ $ml^{-1}$ in RPMI-1640 medium (Nissui, Tokyo, Japan) supplemented with 2 mM L-glutamine, 100 U $ml^{-1}$ penicillin, 100 $\mu g$ $ml^{-1}$ streptomycin, 50 $\mu M$ 2-mercaptoethanol and 5% heat-inactivated human AB serum (Nipro, Osaka, Japan) (complete medium), were used as responder cells (R).

Fresh excised tumour tissues were processed by enzymatic digestion as described previously (Tani *et al.*, 1995; Yamaue *et al.*, 1991; Yamaue *et al.*, 1992; Iwahashi *et al.*, 1992). Briefly, tumour tissues were dissected into pieces smaller than 2 $mm^3$ and these were immersed in medium containing collagenase (2 mg $ml^{-1}$, type V-S; Sigma, St Louis, MO, USA), hyaluronidase (10 U $ml^{-1}$, type IV-S; Sigma) and DNAase-I (0.4 mg $ml^{-1}$; Sigma). After 40 min incubation, cells obtained from solid-tumour specimens and ascites were centrifuged on Ficoll-Hypaque gradients at 400 *g* for 30 min, and the cells were centrifuged on discontinuous gradients consisting of 100% and 75% Ficoll-Hypaque at 400 *g* for 30 min. The tumour-cell-rich fraction was then layered on discontinuous gradients containing 25%, 15% and 10% Percoll (Pharmacia) and centrifuged at 25 *g* for 7 min. Tumour cells depleted of lymphoid cells were collected from the bottom and from the 25% interface, and were then washed and suspended in complete medium. These

Table I Materials and methods

| Case | Diagnosis | Age | Sex | Assay: Proliferation assay | Phenotype | CRA | Cytokine |
|---|---|---|---|---|---|---|---|
| 1 | Thyroid cancer | 81 | F | Yes | No | No | No |
| 2 | Gastric cancer | 62 | F | Yes | Yes | Yes | No |
| 3 | Gastric cancer | 40 | M | Yes | No | No | No |
| 4 | Gastric cancer | 47 | M | Yes | No | No | No |
| 5 | Gastric cancer | 38 | M | No | Yes | Yes | No |
| 6 | Duodenal cancer | 59 | M | Yes | No | Yes | No |
| 7 | Colonic cancer | 61 | F | Yes | Yes | Yes | No |
| 8 | Gallbladder cancer | 56 | F | No | Yes | Yes | Yes |
| 9 | Gallbladder cancer | 59 | F | No | Yes | Yes | Yes |
| 10 | Gallbladder cancer | 70 | F | No | No | No | Yes |
| 11 | Gallbladder carcinosarcoma | 58 | M | Yes | No | No | No |
| 12 | Ovarian cancer | 64 | F | Yes | No | No | Yes |
| 13 | Methothelioma | 53 | M | No | Yes | Yes | Yes |
| 14 | Leiomyosarcoma | 52 | F | Yes | No | Yes | No |
| 15 | Angiosarcoma | 78 | F | Yes | No | No | No |
| 16 | Adenocarcinoma of unknown origin | 71 | M | Yes | No | No | No |

CRA, $^{51}$Cr release assay.

autologous tumour cells, treated with 50 $\mu$g ml$^{-1}$ mitomycin C (MMC; Kyowa Hakko, Tokyo, Japan) at 37°C for 40 min, were used as stimulator cells (S). PBMCs were cultured with autologous tumour cells for 5 days at 37°C in a humidified 5% carbon dioxide atmosphere, at the optimal ratio (PBMC/tumour cells = 100/1) (Tani *et al.*, 1995).

*Preparation of anti-CD3 MAb and fibronectin-coated microtitre plates*

Culture wells were coated for 4 h at room temperature with 10 $\mu$g ml$^{-1}$ of anti-CD3 MAb (muromonab-CD3, $IgG_{2a}$, Orthoclone) in 0.05 M Tris/PBS (pH 9.2) and washed twice with complete medium and once with medium containing 0.5% human AB serum. After washing twice with PBS, 100 $\mu$l ml$^{-1}$ PBS containing the indicated concentrations of human fibronectin (Upstate Biotechnology Inc., USA) was placed in each well and incubated at room temperature for 4 h. Then, the wells were washed twice with complete medium.

*Generation of CTLs induced by AMLTC*

CTLs induced by AMLTC were activated in anti-CD3 MAb-coated wells (with or without immobilised FN) at a concentration of $1 \times 10^5$ per well in complete medium combined with 20, 100, 250 and 1000 IU ml$^{-1}$ IL-2 (S6820; Shionogi, Osaka, Japan) or without IL-2.

*DNA synthesis in CTLs*

DNA synthesis in CTLs was measured by determining [$^3$H]thymidine incorporation ([$^3$H]TdR; NEN, Boston, MA, USA). CTLs ($5 \times 10^5$ ml$^{-1}$) were cultured for 3 days in anti-CD3 MAb-coated flat-bottomed microtitre plates (Falcon 3072; Lincoln, NJ, USA) (with or without immobilised FN) with indicated IL-2 concentration, pulse with 37.5 kBq per well of [$^3$H]TdR during the last 16 h of culture, and harvested. Radioactivity was determined with a liquid scintillator. The experiment was performed in triplicate.

*Cytotoxic assay*

The $^{51}$Cr-release assays (CRA) were performed to assess killer cell cytotoxicity, as described (Iwahashi *et al.*, 1992; Yamaue *et al.*, 1987, 1989). The target cells were used: autologous tumour cells, KATO-III; a gastric carcinoma cell line, and K562; a promyelocytic leukaemia cell line. Target cells were labelled with 3.7 MBq of $Na_2{}^{51}CrO_4$ for 1 h at 37°C and then washed; 100 $\mu$l of $^{51}$Cr-labelled tumour cells ($1 \times 10^5$ ml$^{-1}$) were added to 100 $\mu$l of effector cells in 96-well round-bottomed microtitre plates (Corning, NY, USA, no. 2580). The effector to target ratio was fixed at 15:1, and 30:1, since the data for other ratios were related to data for ratios fixed at 15:1 and 30:1. The experiment was performed in triplicate. After 4 h or 16 h incubation, the radioactivity of the supernatants was determined with a gamma counter (ARC-300, Aloka). The spontaneous release did not exceed 30% for autologous tumour cells and 15% for established tumour cell lines, of maximum release obtained by adding 1 N hydrochloric acid. The background (spontaneous) release of autologous tumour cells was $534 \pm 291$ c.p.m. and $722 \pm 437$ c.p.m. in 4 h and 16 h $^{51}$Cr-release assay respectively. The percentage specific cytotoxicity was calculated as follows (all $^{51}$Cr values in c.p.m.):

$$\frac{\text{Test}^{51}\text{Cr release} - \text{spontaneous release}}{\text{Maximum release} - \text{spontaneous release}} \times 100$$

*Flow cytometric analysis of surface antigens*

The surface antigens of the CTLs were examined by flow cytometry, using FITC- or PE-labelled anti-human CD3, CD4, CD8, CD16, CD25 (Becton-Dickinson, Mountain View, CA, USA) and Mik-$\beta$1 (anti-IL-2-receptor $\beta$-chain, Nichirei, Tokyo, Japan) MAb. The CTLs ($1 \times 10^7$ml$^{-1}$) were suspended in $Ca^{2+}$-$Mg^{2+}$-free PBS (Nissui) containing 0.1% bovine serum albumin (BSA). MAb (100 $\mu$l ml$^{-1}$) was added to the cell suspension, which was then incubated for 30 min at 4°C, washed twice with cold $Ca^{2+}$- and $Mg^{2+}$-free PBS, and resuspended in PBS/0.1% sodium nitrite for flow cytometric examination, including blue laser with an excitation of 15 mW at 488 nm (FACStar, Becton Dickinson). Data were gathered from 10 000 cells using a live gate.

*Analysis of effector cell phenotype by negative selection methods*

Negative selection by immunomagnetic beads was performed as described (Tani *et al.*, 1995; Iwahashi *et al.*, 1992). Activated CTLs ($1 \times 10^7$ ml$^{-1}$) from two patients (one colonic cancer and one gallbladder cancer) were suspended in RPMI-1640 containing immunomagnetic beads coated with 500 $\mu$l ml$^{-1}$ of (M-450; Dynal, Oslo, Norway) sheep IgG, anti-CD4 or anti-CD8 MAb (IgG), and were incubated at 4°C for 30 min with occasional shaking. Rosette-forming cells were then removed with a flat cobalt samarium magnet. Non-rosetted cells were washed, suspended in complete medium containing 10% foetal bovine serum (FBS), and assessed for cytotoxic activity.

*Examination of major-histocompatibility complex restriction*

We determined the major histocompatibility complex (MHC) restriction of the activated CTLs by using MAb. In CRA, anti-HLA-class I MAb (mouse $IgG_{2a}$, IOT 2; Cosmo, Tokyo, Japan), anti-HLA-DR MAb (IOT 2a; Cosmo, Tokyo, Japan) or mouse IgG (Cappel, Organou Technika, Durham, NC, USA) as a control antibody, was added to the microtitre plate, at a final concentration of 5 $\mu g\ ml^{-1}$, which was enough for inhibition in a positive control for these antibodies (Tani *et al.*, 1995; Malissen *et al.*, 1982).

*Cytokine assay in the supernatants*

CTLs ($5 \times 10^5\ ml^{-1}$, 200 $\mu l$ per well), immediately after AMLTC, were cultured in anti-CD3 MAb-coated 96-well flat-bottomed microtitre plates (with or without immobilised fibronectin) at 37°C in a humidified 5% carbon dioxide atmosphere for 24 h without further tumour cell stimulation, and the supernatant was collected after centrifugation. Cytokine activity was assayed by using enzyme-linked immunosorbent assay (ELISA) kits (IL-2, Intertest-2X, Genzyme, Cambridge, MA, USA; IFN-$\gamma$, GM-CSF and IL-4, Medgenix Diagnostics, Fleurus, Belgium; IL-6, Toray-Fuji Bionics, Tokyo, Japan).

*Statistical analysis*

Significant differences were determined by Wilcoxon's test. A $P$-value below 0.05 was considered to be statistically significant. Each value is expressed as the mean ± standard error.

### Results

*Proliferation of CTL by immobilised FN*

As shown in Figure 1, immobilised FN (ranged from 2–50 $\mu g\ ml^{-1}$) augmented the DNA synthesis of CTLs stimulated by immobilised anti-CD3 MAb and IL-2.

Since we have been culturing CTLs stimulated by 10 $\mu g\ ml^{-1}$ immobilised anti-CD3 MAb and 250 IU $ml^{-1}$ IL-2 for adoptive immunotherapy of cancer patients (Tani *et al.*, 1995), we examined the effect of immobilised FN on CTLs stimulated by 10 $\mu g\ ml^{-1}$ immobilised anti-CD3 MAb and 250 IU $ml^{-1}$ IL-2 (Table II). The enhancement of proliferation of CTLs was observed by immobilised FN at concentrations of 2–50 $\mu g\ ml^{-1}$ ($P<0.05$). The proliferative effect of immobilised FN (ranged from 2–50 $\mu g\ ml^{-1}$) was observed at a significant level ($P<0.05$). The proliferative effect of 10 $\mu g\ ml^{-1}$ immobilised FN was significantly higher compared with those of other doses of immobilised FN. Therefore, the dose of 10 $\mu g\ ml^{-1}$ FN was used for all subsequent studies.

Moreover, the cell numbers of CTLs stimulated by immobilised FN was three or four times higher than CTLs stimulated without FN (Figure 2). On the other hand, the soluble FN did not alter the proliferation and cell numbers of CTLs stimulated by immobilised anti-CD3 MAb and 250 IU $ml^{-1}$ of IL-2 (data not shown).

**Figure 1** Dose titration of immobilised fibronectin on the proliferation of CTL. CTL ($5 \times 10^5 ml^{-1}$) were stimulated with immobilised fibronectin (ranging from 2 to 50 $\mu g\ ml^{-1}$), immobilised anti-CD3 MAb (10 $\mu g\ ml^{-1}$) and IL-2 (ranging from 0 to 1000 IU $ml^{-1}$). Control means CTL stimulated without fibronectin and anti-CD3 MAb.

*Cytotoxicity of CTLs generated by immobilised FN*

In all cases that we could examine, the cytotoxic activity of CTLs was augmented by FN stimulation against autologous tumour cells, by 4 h and 16 h CRA respectively. On the other hand, the cytotoxic activity of CTLs against other established tumour cell lines did not show a tendency to be augmented by FN stimulation (Table III).

*Surface phenotypes of CTLs generated by immobilised FN*

The phenotypes of the CTLs (two gastric cancer, one colonic cancer, one gallbladder cancer and one malignant methothelioma of the peritoneum) were determined by flow cytometry. The major cell surface phenotype of FN-stimulated CTLs was $CD3^+$, $CD4^+$ and $CD25^+$ (Table IV).

*MHC restriction of CTLs generated by immobilised FN*

As shown in Table V, the autologous tumour killing activity was suppressed by anti-HLA class I MAb, whereas it was not altered by anti-HLA-DR MAb. These findings indicate that HLA class I restricted CTLs were generated by immobilised FN. We examined the phenotypes of these CTLs by a negative selection method employing immunomagnetic

**Table II** DNA synthesis of CTL

| Case | Control | FN ($\mu g\,ml^{-1}$) 0 | 2 | 5 | 10 | 50 |
|---|---|---|---|---|---|---|
| Case 1 | 45 638 | 68 884 | 76 328 | 73 877 | 74 082 | 73 854 |
| Case 2 | 11 576 | 31 985 | 35 055 | 35 625 | 35 156 | 34 605 |
| Case 3 | 92 191 | 193 500 | 207 787 | 201 810 | 220 029 | 213 013 |
| Case 4 | 76 113 | 104 794 | 116 151 | 124 126 | 122 337 | 118 399 |
| Case 6 | 57 598 | 119 668 | 145 127 | 156 815 | 156 404 | 149 251 |
| Case 7 | 23 705 | 35 499 | 39 090 | 38 657 | 40 341 | 40 065 |
| Case 15 | 30 094 | 55 542 | 65 896 | 67 479 | 68 798 | 68 573 |
| Case 16 | 12 843 | 33 838 | 42 841 | 46 603 | 46 900 | 43 314 |
| Mean | 43 719 | 80 457 | 91 034* | 93 124* | 95 506* | 92 009* |
| s.e. | 10 494 | 19 914 | 21 626 | 21 669 | 23 205 | 22 407 |

[$^{3}$H]Td R incorporation (c.p.m.). CTLs ($5\times10^{5}\,ml^{-1}$) were stimulated with immobilised fibronectin (ranged from 2–50 $\mu g\,ml^{-1}$), immobilised anti-CD3 MAb (10 $\mu g\,ml^{-1}$) and IL-2, 250 IU $ml^{-1}$. Control shows CTL stimulated without fibronectin and anti-CD3 MAb. *$P<0.05$, compared with control and FN, 0 $\mu g\,ml^{-1}$.

**Figure 2** The growth curve of CTL. The cell numbers were counted in three patients. The cell numbers at the start of culture were $3\times10^{6}$. ●, CTL stimulated with immobilised fibronectin, immobilised anti-CD3 MAb and IL-2, 250 IU $ml^{-1}$. ○, CTL stimulated without immobilised fibronectin.

**Table III** Cytotoxicity of activated CTL

| | Cx(%)[a] AUTO 4h | | AUTO 16h | | K562 4h | | K562 16h | | KATO-III 4h | | KATO-III 16h | |
|---|---|---|---|---|---|---|---|---|---|---|---|---|
| FN stimulation | (−) | (+) | (−) | (+) | (−) | (+) | (−) | (+) | (−) | (+) | (−) | (+) |
| Case 2 (day 42) | 25.4 | 40.5 | | | | | | | 39.5 | 47.5 | 53.4 | 61.1 |
| Case 5 (day 21) | 5.1 | 8.2 | 8.8 | 17.8 | 2.0 | 1.7 | 8.3 | 4.9 | 18.6 | 17.3 | 48.9 | 45.3 |
| Case 6 (day 21) | 8.1 | 8.6 | 7.8 | 15.8 | | | | | 33.2 | 37.1 | 39.6 | 41.4 |
| Case 7 (day 42) | 6.6 | 10.6 | 27.7 | 34.0 | | | | | 26.9 | 33.0 | 34.9 | 35.5 |
| Case 8 (day 28) | 5.9 | 8.2 | 13.2 | 22.8 | 4.3 | 4.3 | 11.6 | 11.6 | 26.2 | 23.9 | 64.2 | 58.2 |
| Case 9 (day 28) | 3.0 | 13.6 | 20.6 | 26.5 | 15.0 | 11.5 | 31.1 | 27.2 | 24.5 | 24.2 | 53.9 | 46.1 |
| Case 13 (day 28) | 22.0 | 28.3 | | | 2.8 | 1.9 | | | 6.2 | 5.3 | 24.6 | 24.1 |
| Case 14 (day 21) | 9.5 | 12.9 | | | 5.3 | 4.6 | | | 5.5 | 9.8 | 31.3 | 24.8 |
| Mean | 10.7 | 16.4* | 15.6 | 23.4* | 5.8 | 4.8 | 17.0 | 14.5 | 22.5 | 24.7 | 43.8 | 42.0 |
| s.e. | 2.9 | 4.1 | 3.7 | 3.2 | 2.3 | 1.7 | 7.1 | 6.6 | 4.2 | 4.9 | 4.7 | 4.8 |

The cytotoxic activity of CTL against autologous tumours was augmented by FN stimulation (*$P<0.05$). Activated CTL were generated by stimulation with 10 $\mu g\,ml^{-1}$ immobilised anti-CD3 MAb, 250 IU $ml^{-1}$ IL-2 with or without 10 $\mu g\,ml^{-1}$ immobilised fibronectin. [a]Cytotoxicity was measured by 4 h and 16 h $^{51}$Cr-release assay at the ratio of 15:1 in triplicate.

Table IV Phenotypic analysis of activated CTL

| | (Day) | Stimulation | Positive cells (%) CD3 | CD4 | CD8 | CD16 | CD25 | IL-2Rβ |
|---|---|---|---|---|---|---|---|---|
| Case 5 | (10) | CD3 | 84.4 | 33.3 | 56.7 | 0.5 | 45.4 | 0.7 |
| | | CD3+FN | 81.9 | 31.7 | 53.8 | 0.7 | 41.9 | 0.5 |
| | (21) | CD3 | 98.4 | 75.6 | 20.5 | 0.4 | 57.3 | 0.8 |
| | | CD3+FN | 94.5 | 73.6 | 24.7 | 0.3 | 62.0 | 0.5 |
| Case 9 | (21) | CD3 | NT | NT | NT | NT | NT | NT |
| | | CD3+FN | 97.5 | 80.9 | 17.8 | 0.02 | 65.2 | 0.21 |
| Case 8 | (28) | CD3 | 91.3 | 79.4 | 1.4 | 0.6 | 28.8 | 0.6 |
| | | CD3+FN | 85.9 | 85.5 | 12.1 | 0.5 | 32.9 | 0.4 |
| Case 13 | (28) | CD3 | 94.9 | 95.8 | 4.3 | 0.3 | 43.3 | 0.1 |
| | | CD3+FN | 91.6 | 91.9 | 8.0 | 0.5 | 35.2 | 0.1 |
| Case 7 | (42) | CD3 | NT | NT | NT | NT | NT | NT |
| | | CD3+FN | 99.3 | 98.1 | 1.5 | 0.53 | 90.1 | 0.43 |
| Case 2 | (42) | CD3 | NT | NT | NT | NT | NT | NT |
| | | CD3+FN | 99.0 | 97.7 | 0.7 | 0.01 | 92.1 | 0.36 |

The phenotypes of the activated CTL were examined by flow cytometry in two gastric cancer (cases 2 and 5), one colonic cancer (case 7), two gallbladder cancer (cases 8 and 9) and one malignant methothelioma of peritoneum (case 13). NT, not tested.

Table V MHC restriction and effector cells of CTL against autologous tumour cells

| | Treatment of CTL | Autologous tumour-killing activity (% Cx) E/T=15 | E/T=30 |
|---|---|---|---|
| | Media | 11.6 | 14.9 |
| | Mouse IgG | 11.3 | 14.9 |
| Case 7 | Anti-CD4 | 3.1 | 5.8 |
| (day 42) | Anti-CD8 | 10.6 | 14.7 |
| | Anti-HLA class I | 2.7 | 9.2 |
| | Anti-HLA-DR | 10.5 | 14.2 |
| | Media | 8.5 | |
| | Mouse IgG | 7.6 | |
| Case 8 | Anti-CD4 | 0.1 | |
| (day 28) | Anti-CD8 | 8.7 | |
| | Anti-HLA class I | 4.4 | |
| | Anti-HLA-DR | 8.1 | |

Phenotypes of CTL activated by immobilised FN were determined by negative selection method employing immunomagnetic beads separation. In blocking assay, 5 $\mu g ml^{-1}$ anti-HLA class I MAb or anti-HLA-DR MAb was used. The autologous tumor-killing activity was determined by 4 h $^{51}Cr$-release assay. CTL were induced from patients with one colonic cancer (case 7) and one gallbladder cancer (case 8).

Table VI Cytokine production by CTL

| FN stimulation | IL-2 ($pg ml^{-1}$) (−) | (+) | IFN-γ ($pg ml^{-1}$) (−) | (+) | GM-CSF ($pg ml^{-1}$) (−) | (+) | IL-4 ($pg ml^{-1}$) (−) | (+) | IL-6 ($pg ml^{-1}$) (−) | (+) |
|---|---|---|---|---|---|---|---|---|---|---|
| Case 8 | 0 | 823 | 11 | 41 | 31 | 464 | 5 | 14 | 236 | 306 |
| Case 9 | 282 | 316 | 10 | 46 | 49 | 2156 | 11 | 97 | 135 | 158 |
| Case 10 | 162 | 698 | 0 | 29 | 61 | 340 | 0 | 27 | 772 | 1362 |
| Case 12 | 50 | 75 | 7 | 30 | 59 | 70 | 0 | 0 | 395 | 480 |
| Case 13 | 318 | 380 | 1 | 8 | 714 | 3112 | NT | NT | NT | NT |

Cytokine production by CTL was measured. CTL were induced from the three patients with gallbladder cancer (cases 8–10), one ovarian cancer (case 12) and one malignant methothelioma of peritoneum (case 13). Cytokine production including IL-2, IFN-γ and GM-CSF was augmented by FN stimulation (*$P<0.05$).

separation. The autologous tumour killing activity of these CTLs was suppressed by the elimination of $CD4^+$ cells. These results indicate that $CD4^+$ CTLs are strongly cytotoxic against autologous tumour cells.

### Cytokine activity produced by CTLs

The cytokine production by CTLs immediately after AMLTC induced from five patients (three gallbladder cancer, one ovarian cancer and one malignant methothelioma of the peritoneum) was studied. As shown in Table VI the cytokine production of CTLs was augmented by FN stimulation. In particular, the production of IL-2, IFN-γ and GM-CSF was significantly augmented by FN stimulation ($P<0.05$).

### Discussion

Rosenberg *et al.* (1987, 1988) have reported that the therapy with LAK cells and IL-2 showed 30% response in renal cell cancer, reduced to 17% in follow-up studies, and TILs plus

IL-2 showed 50% response in melanoma, although long-term follow-up to determine durability of response has not been published.

We have reported the basic study of AIT using TILs activated by IL-2 and IL-4 that exhibited high autologous tumour killing activity and high proliferative response (Yamaue *et al.*, 1990; Tsunoda *et al.*, 1992).

However, TILs cannot be induced in all cancer patients. Therefore, we have chosen CTL induction by AMLTC to overcome the problems of using TILs in AIT. Although AIT using CTLs has been shown to be effective in clinical trials, CTLs induced by AMLTC are not sufficiently proliferated by IL-2 alone (Maeda *et al.*, 1989; Aruga *et al.*, 1991). We have reported that CTLs can be sufficiently proliferated by immobilised anti-CD3 MAb and IL-2 (Tani *et al.*, 1995). However, finding methods by which CTLs can be proliferated more extensively is an important research area. The extracellular matrix (ECM) is composed of a number of macromolecules that promote cell adhesion, cell migration and differentiation. Recently, ECM, such as FN, laminin and collagen, has been reported to co-stimulate the DNA synthesis of human PBLs (Shimizu *et al.*, 1990; 1990; Matsuyama *et al.*, 1989). It has been reported that FN induces AP-1 as an enhancer of IL-2 gene with signal transduction of FN receptor (Yamada 1991).

Although FN alone has no ability to induce proliferation of human PBLs, FN augments the proliferation of human PBLs induced by immobilised anti-CD3 MAb, and the effect of FN is stronger than any other ECM (Davis *et al*,. 1990; Cardarelli *et al.*, 1991). However, there has been no report of FN on the proliferation and activation of CTLs induced by AMLTC from cancer patients.

In the present study, we examined whether FN promotes the proliferation and activation of CTLs generated by immobilised anti-CD3 MAb and IL-2. DNA synthesis of CTLs induced by AMLTC was augmented by immobilised FN. Moreover, CTLs stimulated by immobilised FN showed three or four times the proliferative response compared with CTLs stimulated without FN in 3 weeks' culture. Since CTLs induced by AMLTC are hard to proliferative by IL-2 alone, the leukapheresis has been required to obtain PBMCs from the cancer patients for AIT using CTLs (Aruga *et al.*, 1991). The present study indicates that CTLs can show marked proliferation by the stimulation with immobilised FN.

Furthermore, for improvement of clinical efficacy, CTLs are required to have higher autologous tumour killing activity. In our present study, CTLs stimulated by immobilised FN exhibited higher autologous tumour killing activity than CTLs stimulated without FN. However, cytotoxic activity against the NK-sensitive tumour cell line, K562, or the NK-resistant tumour cell line, KATO-III, was not augmented by FN stimulation. These results indicate that the specific cytotoxic activity against autologous tumour cells was augmented by FN stimulation.

We have reported that the surface phenotypes of CTLs activated with immobilised anti-CD3 MAb and IL-2 were predominantly $CD3^+$ and $CD4^+$ (Tani *et al.*, 1995). In the present study, CTLs activated with immobilised FN showed more predominantly $CD4^+$ T cells, and the autologous tumour killing activity was suppressed by the elimination of $CD4^+$ CTLs.

Two types of mouse $CD4^+$ Th cell clones differ in their lymphokine production pattern (Mosmann *et al.*, 1986). It has been reported that the restricted cytokine profiles exist in human $CD4^+$ T-cell populations (Maggi *et al.*, 1991). Thl cells secrete IL-2, IFN-$\gamma$ and lymphotoxin, but they do not secrete IL-4, IL-5 or IL-6. On the other hand, Th 2 cells secrete IL-4, IL-5 and IL-6, but they do not produce IL-2, IFN-$\gamma$ or lymphotoxin. Moreover, it has been reported that Th 0 cells, which secreted a broad spectrum of cytokines, including IL-2, IL-3, IL-4, IFN-$\gamma$ and TNF, were found (Firestein *et al.*, 1989; Mosmann *et al.*, 1991). Since we studied the cytokine production profiles in bulk culture CTLs, not in CTL clones, we could not find the difference of response between Thl, Th2 and Th0 by FN stimulation. However, immobilised FN up-regulated the production of IL-2, IFN-$\gamma$ and GM-CSF in bulk culture. The results suggested that FN might stimulate the activity of TH1 cells, and up-regulated cytokine production might induce the strong autologous tumour killing activity.

CTLs stimulated by IL-2 alone appear to be $CD8^+$ cell and MHC class I restricted, since their cytotoxicity has been shown to be inhibited by anti-HLA class I MAb and by elimination of $CD8^+$ cells (Sato *et al.*, 1986; Maeda *et al.*, 1989; Aruga *et al.*, 1991; Ioannides *et al.*, 1991). However, we have reported that $CD4^+$ CTLs generated by immobilised anti-CD3 MAb and IL-2 are involved in the cytotoxicity against autologous tumour cells and restricted by HLA-DR (Tani *et al.*, 1995). In the present study, we demonstrated that $CD4^+$ T cells activated with immobilised FN, were strongly cytotoxic against autologous tumours, and restricted by HLA class I, not by HLA-DR. Matsubayashi *et al.* (1989) have demonstrated that the specific $CD4^+$ CTL clone for Friend virus-induced FBL-3 tumour cells is restricted by HLA class I.

Moreover, in human systems, the auto-killing activity of $CD4^+$ CTL clone specific for human gastric cancer cells and human melanoma cells is restricted by HLA class I (Itoh *et al.*, 1992; Wang *et al.*, 1992). In the present study, we studied the HLA restriction only in bulk culture CTLs. Further studies are considered to be necessary to clarify the HLA-restriction of CTLs stimulated by FN.

$CD4^+$ T cells have been shown to be more susceptible to immunosuppressive effects in the tumour-bearing state than $CD8^+$ T cells (Tada *et al.*, 1990). Improving the immunosuppressive state in cancer patients requires recovery of helper function on $CD4^+$ T cells. Therefore, infusing $CD4^+$ T cells, which have helper function, is essential in improving the clinical efficacy in AIT for cancer patients.

Thus, our results indicate that immobilised FN promotes the proliferation and the activation of CTLs, and FN-activated CTLs might be effective for adoptive immunotherapy in cancer patients.

**Acknowledgements**

This work was supported in part by a grant-in-aid from the Ministry of Education, Science and Culture of Japan, by grants-in-aid from the Japanese Foundation for multidisciplinary treatment of cancer and by grants-in-aid from Fujita Memorial Fund for Medical Research. We wish to thank Ms Mariko Sugano for her excellent technical assistance.

### References

ARUGA A, YAMAUCHI K, TAKASAKI K, FURUKAWA T AND HANYU F. (1991). Induction of autologous tumour-specific cytotoxic T cells in patients with liver cancer. Characterizations and clinical utilization. *Int. J. Cancer*, **49**, 19–24.

CARDARELLI PM, YAMAGATA S, SCHOLZ W, MOSCINSKI MA AND MORGAN EL. (1991). Fibronectin augments anti-CD3-mediated IL-2 receptor (CD25) expression on human peripheral blood lymphocytes. *Cell. Immunol.*, **135**, 105–117.

DAVIS LS, OPPENHEIMER-MARKS N, BEDNARCZYK JL, MACINTYRE BW AND LIPSKY PE. (1990). Fibronectin promotes proliferation of nave and memory T cells by signaling through both the VLA-4 and VLA-5 integrin molecules. *J. Immunol.*, **145**, 785–793.

FIRESTEIN GS, ROEDER WB, LAXER JA, TOWNSEND KS, WEAVER CT, HOM JT, LINTON J, TORBETT BE AND GLASEBROOK AL. (1989). A new murine CD4$^+$ T cell subset with an unrestricted cytokin profile. *J. Immunol.*, **143,** 518–525.

IOANNIDES CG, RASHED S, FISK B, FAN D, ITOH K AND FREEDMAN RS. (1991). Lymphocytes infiltrating ovarian malignant ascites: modulation of IL-2-induced proliferation by IL-4 and of selective increase in CD8$^+$ T cells by TNF-$\alpha$. *Lymphokine Cytokine Res.*, **10,** 307–315.

ITOH K, SALMERON MA, MORITA T, SEITO D, MANSFIELD PF, ROSS MI, BALCH CM AND AUGUSTUS LB. (1992). Distribution of autologous tumour-specific cytotoxic T lymphocytes in human metastatic melanoma. *Int. J. Cancer,* **52,** 52–59.

IWAHASHI M, TANIMURA H, YAMAUE H, TSUNODA T, TANI M, TAMAI M, NOGUCHI K AND HOTTA T. (1992). Defective autologous mixed lymphocyte reaction (AMLR) and killer activity generated in the AMLR in cancer patients. *Int. J. Cancer*, **51,** 67–71.

MAEDA N, HASHIMOTO S, MIYAZAKI H, TAKATA M, YAMAMOTO H AND FUJIMOTO S. (1989). Augmentation of human cytotoxic T lymphocytes against autologous tumour by a factor release from human monocytic leukemia cell line. *Jpn. J. Cancer Res.*, **80,** 537–545.

MAGGI E, BISWAS P, PRETE GD, PARRONCHI PP, MACCHIA D, SIMONELLI C, EMMI L, CARLI MD, TIRI A, RICCI M AND ROMAGNANI S. (1991). Accumulation of Th-2-like helper T cells in the conjunctiva of patients with vernal conjunctivitis. *J. Immunol.*, **146,** 1169–1174.

MALISSEN B, REBAI N, LIABEUF A AND MAWAS C. (1982). Human cytotoxic T-cell structures associated with expression of cytolysis. I. Analysis at the clonal cell level of the cytolysis-inhibiting effect of 7 monoclonal antibodies. *Eur. J. Immunol.*, **12,** 739–747.

MATSUBAYASHI Y, ZENITA K, MORIOKA A, IWASHIRO M, MASUDA T, UCHINO H, FUJITA T AND KURIBAYASHI K. (1989). Characterization of a CD4(L3T3)-positive cytotoxic T cell clone that is restricted by class I major histocompatibility complex antigen on FBL-3 tumour cell. *Immunobiology*, **180,** 33–46.

MATSUYAMA T, YAMADA A, KAY J, YAMADA KM, AKIYAMA SK, SCHLOSSMAN SF AND MORIMOTO C. (1989). Activation of CD4 cells by fibronectin and anti-CD3 antibody. A synergistic effect mediated by the VLA-5 fibronectin receptor complex. *J. Exp. Med.*, **170,** 1133–1148.

MOSMANN TR, CHERWINSKI H, BOND MW, GIEDLIN MA AND COFFMAN RL. (1986). Two types of murine helper T cell clone I. Definition according to profiles of lymphokine activities and secreted proteins. *J. Immunol.*, **136,** 2348–2357.

MOSMANN TR, SCHUMACHER JH, STREET NF, BUDD R, O'GARRA A, FONG TA, BOND MW, MOORE KWM, SHER A AND FIORENTINO DF. (1991). Diversity of cytokine synthesis and function of mouse CD4$^+$ T cells. *Immunol. Rev.*, **123,** 209–229.

ROSENBERG SA, LOTZE MT, MUUL LM, CHANG AE, AVIS FP, LEITMAN S, LINEHAN WM, ROBERTSON CN, LEE RE, RUBIN JT, SEIPP CA, SIMPSON AND WHITE DE. (1987). A progress report on the treatment of 157 patients with advanced cancer using lymphokine-activated killer cells and interleukin-2 or high-dose interleukin-2 alone. *N. Engl. J. Med.*, **316,** 889–897.

ROSENBERG SA, PACKARD BS, AEBERSOLD PM, SOLOMON D, TOPALIAN SL, TOY ST, SIMON P, LOTZE MT, YANG JC, SEIPP CA, SIMPSON CG, CARTER C, BOCK S, SCHWARTZENTRUBER D, WEI JP AND WHITE DE. (1988). Use of tumour-infiltrating lymphocytes and interleukin-2 in the immunotherapy of patients with metastatic melanoma. *N. Eng. J. Med.*, **319,** 1676–1680.

SATO T, SATO N, TAKAHASHI S, KOSHIBA H AND KIKUCHI K. (1986). Specific cytotoxicity of a long-term cultured T-cell clone on human autologous mammary-cancer cells. *Cancer Res.*, **46,** 4384–4389.

SHIMIZU Y, VAN SEVENTER GA, HORGAN KJ AND SHAW S. (1990). Costimulation of proliferative responses of resting CD4 T cells by the interaction of VLA-4 and VLA-5 with fibronectin or VLA-6 with fibronectin or VLA-6 with laminin. *J. Immunol.*, **145,** 59–67.

SHIMIZU Y, VAN SEVENTER GA, HORGAN KJ AND SHAW S. (1990). Roles of adhesion molecules in T-cell recognition: fundamental similarities between four integrins on resting human T cells (LFA-1, VLA-4, VLA-5, VLA-6) in expression, binding, and costimulation. *Immunol. Rev.*, **114,** 109–143.

TADA T, SANO H, SATO S, SHIMA J, FUJIWARA H AND HAMAOKA T. (1990). Immune dysfunction expressed selectively on L3T4$^+$ T cells in the tumour-bearing state. *J. Leukocyte Biol.*, **47,** 149–157.

TANI M, TANIMURA H, YAMAUE H, MIZOBATA S, IWAHASHI M, TSUNODA T, NOGUCHI K, TAMAI M, HOTTA T, TERASAWA H AND ARII K. (1995). Generation of CD4$^+$ cytotoxic T lymphocytes stimulated by immobilized anti-CD3 monoclonal antibody and interleukin-2 in cancer patients. *Int. J. Cancer*, **60,** 802–807.

TSUNODA T, TANIMURA H, YAMAUE H, IWAHASHI M, TANI M, TAMAI M, ARII K AND NOGUCHI K. (1991). *In vitro* augmentation of the cytotoxic activity of peripheral-blood mononuclear cells and tumour infiltrating lymphocytes by famotidine in cancer patients. *Int. J. Immunopharmacol.*, **14,** 75–81.

TSUNODA T, TANIMURA H, YAMAUE H, IWAHASHI M, TANI M, TAMAI M, ARII K AND NOGUCHI K. (1992). The promotive effect of interleukin 4 with interleukin 2 in the proliferation of tumour-infiltrating lymphocytes from patients with malignant tumour. *Biotherapy*, **4,** 9–15.

WANG P, VANKY F AND KLEIN E. (1992). MHC class-I-restricted auto-tumour-specific CD4$^+$CD8$^-$ T-cell clones established from autologous mixed lymphocyte-tumour-cell culture (MLTC). *Int. J. Cancer*, **51,** 962–967.

YAMADA A, NIKAIDO T, NOJIMA Y, SCHLOSSMAN SF AND MORIMOTO C. (1991). Activatin of human CD4 T lymphocytes. Interaction of fibronectin with VLA-5 receptor on CD4 cells induces the AP-1 transcription factor. *J. Immunol.*, **146,** 53–56.

YAMAUE H, KATSUMI M, TABUSE K, TABUSE Y, KURIBAYASHI K AND SAITO K. (1987). Induction of activated natural killer cells from murine spleen cells primed *in vivo* and subsequently challenged *in vitro* with the streptococcal preparation OK432. *Cancer Immunol. Immunother.*, **25,** 169–174.

YAMAUE H., TANIMURA H, IWAHASHI M, TANI M, TSUNODA T, TABUSE K, KURIBAYASHI K AND SAITO K. (1989). Role of interleukin-2 and interferon-$\gamma$ induction of activated natural killer cells from mice primed *in vivo* and subsequently challenged in vitro with the streptococcal preparation OK432. *Cancer Immunol. Immunother.*, **29,** 79–86.

YAMAUE H, TANIMURA H, TSUNODA T, IWAHASHI M, TANI M, TAMAI M AND INOUE M. (1990). Functional and phenotypic analyses of interleukin-2 activated tumour-infiltrating lymphocytes. *Biotherapy*, **2,** 247–259.

YAMAUE H, TANIMURA H, TSUNODA T, TANI M, IWAHASHI M, NOGUCHI K, TAMAI M, HOTTA T AND ARII K. (1991). Chemosensitivity testing with highly purified fresh human tumour cells using the MTT colorimetric assay. *Eur. J. Cancer*, **27,** 1258–1263.

YAMAUE H, TANIMURA H, NOGUCHI K, HOTTA T, TANI M, TSUNODA T, IWAHASHI M, TAMAI M AND IWAKURA S. (1992). Chemosensitivity testing of fresh human gastric cancer with highly purified tumour cell using the MTT assay. *Br. J. Cancer*, **66,** 794–799.